# Long-Term Use of a Sensory Prosthesis Improves Function in a Patient With Peripheral Neuropathy: A Case Report

**DOI:** 10.3389/fneur.2021.655963

**Published:** 2021-06-23

**Authors:** Diane M. Wrisley, Gillian McLean, Jennifer Baity Hill, Lars I. E. Oddsson

**Affiliations:** ^1^Department of Physical Therapy, Wingate University, Wingate, NC, United States; ^2^Doctor of Physical Therapy Program, College of St Mary, Omaha, NE, United States; ^3^Fyzical Therapy and Balance Centers, Las Vegas, NV, United States; ^4^OrthoCarolina Outpatient Winston-Salem, Winston-Salem, NC, United States; ^5^Department of Rehabilitation Medicine, Medical School, University of Minnesota, Minneapolis, MN, United States; ^6^Recanati School of Community Health, Ben Gurion University of the Negev, Beersheba, Israel; ^7^RxFunction Inc., Eden Prairie, MN, United States

**Keywords:** balance, sensory substitution, neuroprosthesis, peripheral neuropathy, fall risk, diabetes, case report, gait

## Abstract

**Background:** Peripheral neuropathy (PN) can result in either partial or complete loss of distal sensation resulting in an increased fall risk. Walkasins® uses a shoe insert to detect the magnitude and direction of sway and sends signals to a leg unit that provides sensory balance cues. The objective of this case report is to describe the long-term influence of the Walkasins® lower limb sensory neuroprosthesis on balance and gait for an individual with diabetic PN.

**Case Description:** A 51-year-old male with a 3-year history of PN and a 10-year history of type II diabetes mellitus was fitted bilaterally with Walkasins® and utilized them 8–10 hours/day for more than 2 years. Although, vibration and tactile sensation thresholds were severely impaired at his 1st metatarsophalangeal joint and the lateral malleolus bilaterally he could perceive tactile stimuli from the Walkasins® above the ankles.

**Outcomes:** Following Walkasins® use, his Activities-specific Balance Confidence Scale (ABC) scores improved from 33 to 80%. His mean Vestibular Activities of Daily Living (VADL) scores decreased from 3.54 to 1. His Functional Gait Assessment (FGA) scores increased from 13/30 to 28/30 and his miniBESTest scores improved from 15/28 to 26/28. Gait speed increased from 0.23 to 1.5 m/s. The patient described a decrease in pain and cramping throughout his lower extremities and an increase in function.

**Discussion:** Gait and balance improved with the use of the Walkasins® and participation in a wellness program. This improvement suggests that the use of sensory substitution devices, such as the Walkasins®, may replace sensory deficits related to gait and balance dysfunction experienced by patients with PN. Further research is needed to determine if other patients will have a similar response and what the necessary threshold of sensory function is to benefit from use of the Walkasins®.

## Background and Purpose

Approximately 12% of the adult population in the United States has been diagnosed with diabetes mellitus with the prevalence in adults 45–64 being 17% (1). The prevalence is rising due to aging, obesity and decreases in physical activity (2). Thirty to fifty percent of adults with diabetes present with peripheral neuropathy (PN) (3–6). The number of people with PN may be higher as many cases are subclinical, undiagnosed, or are underreported (2, 7). Peripheral neuropathy can be either motor, autonomic, sensory or a combination of all three (8). The majority of people with PN experience autonomic neuropathy with the second most common form being sensory neuropathy. With diabetes, the most common type of PN is sensory (9). Symptoms of PN include autonomic, motor, and sensory dysfunction resulting in balance and gait impairments. People with PN demonstrate increased sway with their eyes closed and more sway response to visual flow (10, 11). Decreased use of an ankle strategy is also noted as is decreased reaction time which both can increase the risk of falling (12).

Gait speed and stride length are decreased in people with diabetes (13, 14). Persons with PN demonstrate a wide base of support, greater step time variability, greater time spent in double limb support, and impaired pressure distribution of the foot (14, 15). People with painful PN demonstrate greater variability in step length and velocity (13). Ankle mobility and power are decreased as well (16). It is thought that the sensory changes that occur with PN contribute to gait impairments (13).

People with PN have an increased risk of falling (14, 17, 18). Approximately 29% of people with diabetic peripheral neuropathy have experienced a fall in the previous 12 months (19). Peripheral neuropathy is significantly associated with falling and repetitive falling. Fall risk increases when the person is in pain or experiencing gait and balance dysfunction (20). Most falls occur during gait (14, 17, 21).

Although current physical therapy interventions can improve balance and gait function in people with PN, the improvements are only minimal. In two systematic reviews of interventions for diabetic peripheral neuropathy, lower extremity strengthening was given only a fair recommendation (22, 23). All other interventions, including electrotherapy (22) and monochromatic light therapy (22), had insufficient evidence to recommend them for decreasing pain and increasing function (24). Salsabili et al. (24), however, found an improvement in Timed “Up and Go” scores and Falls Efficacy Scale scores following 4 weeks of task-oriented gait and balance training in a case series. Timed “Up & Go” scores decreased greater than the Minimal Detectable Change (MDC) from an average of 10–7.3 s (25).

The use of plantar electrical stimulation daily for 6 weeks improved gait (stride velocity and stride time) and balance parameters (sway eyes open) as well as vibratory plantar threshold when compared to a control group that also improved but less than the intervention group (26). Gait speeds increased from 0.87 to 0.97 m/s in the intervention group and from 0.82 to 0.90 m/s in the control group. Considering such marginal changes in outcomes, interventions that can improve gait and balance function in people with PN is needed. The SENSUS device used in the Najafi et al. (26) study was a TENS unit applied to the plantar surface for an hour each night.

Vibrotactile sensory substitution devices have been used experimentally to improve gait and balance function in people with various sensory deficits including older adults at risk for falls and vestibular dysfunction (27–34). Sensory substitution devices may use accelerometers and gyroscopes to replace lost vestibular function or foot pressure sensors to replace lost plantar mechanoreceptor function to help determine the body position in space and improve balance (31). Furthermore, pilot data suggests a possible augmentation effect where gait and balance function may improve although the sense appears clinically intact (e.g., balance-related foot pressure information may enhance balance in patients with vestibulopathy although plantar sensation is intact, and/or vestibular information may enhance balance in PN patients) (27, 31, 34).

Previously, sensory substitution devices have been used for short periods of time, usually up to 1 month (35). Improvements have been demonstrated with carryover up to 6 months (32) but the use of a device long term as a balance prosthesis to improve gait and balance in people with peripheral neuropathy had not been investigated. Recently we published a paper regarding the 10 week use of Walkasins in people with PN. The subjects improved gait and balance function as demonstrated by improvements in the Functional Gait Assessment (FGA), Timed “Up & Go,” gait speed, and a decrease infall rate (36). The subjects were not allowed to participate in any other form of balance intervention during the 10 weeks of the study. The purpose of this case report is to describe improvements in gait and balance seen in a patient with diabetic PN when wearing Walkasins®, a sensory prosthesis, long term (6–8 h/day) for more than 2 years. It is unique from the previous paper as it demonstrates that improvements were seen with the device as well as introducing the role of rehabilitation with the device. The device provides directional specific tactile stimuli based on measurements of plantar center of pressure with a sensor embedded insole placed in the shoe. In a recent randomized cross-over trial, (37) short-term in clinic improvements in both FGA and gait speed associated with wearing the Walkasins® device turned on were demonstrated in a group of patients with PN. The frequency of the tactile stimuli provided by the device is in the range of the clinically used 128 Hz tuning fork, although the stimulus amplitude is higher. Consequently, patients who may be insensate to the tuning fork may still be able to feel the device stimulus. We hypothesize that the device can replace lost foot pressure sensation with new sensory balance signals that modulate skin mechanoreceptors using tactile stimulators on the calf proximal to the nerve lesion where the patient's sensation is still sufficiently intact (31, 37).

## Case Description: Patient History and Systems Review

The patient was a 51-year-old male who was referred for physical therapy due to balance and gait difficulties secondary to diabetic PN. He was referred to the Neurologic Wellness Program at Wingate University. The Neurological Wellness Program is a student run (pro-bono) clinic that provides exercise, gait, and balance training to people with neurological disorders who do not have health insurance or have exhausted their insurance.

His primary complaint was that his feet were numb. His past medical history included a 10-year history of type II Diabetes Mellitus, well-controlled with medication, mild back pain, peripheral neuropathy, high blood pressure, and kidney failure. He denied any falls in the past year. He complained of a not being able to feel his feet, walking on bricks, tingling, and pain. He had a diagnosis of primary sensory PN with moderate pain and generalized weakness. Prior to the diagnosis of PN, he was very active playing 20 h of tennis per week, roller blading and playing basketball. He was a truck driver for many years prior to his diagnosis and had to stop driving as he could not feel his feet.

He was alert and oriented, appeared cognitively intact and was a good historian. His medication included 81 mg aspirin, sertraline, Lisinopril, meclizine (due to generalized dizziness), pravastatin, chlorpromazine, Lyrica, omeprazole, Apidra, Lantus, iron, vitamin C, B12, and D3. His blood pressure was 150/98 with O2 saturation of 98%. His integumentary system was normal as was his range of motion except for ankle dorsiflexion to neutral bilaterally. Strength was generally 4/5 with the exception of ankle dorsiflexion which was 3+/5 bilaterally. He ambulated ~0.25 miles in the community with a straight cane and was independent in all mobility and ADLs. His light touch and sharp/dull sensation was impaired below the knee bilaterally. He was unable to detect the 50 g monofilament below the knee but was able to detect the 2 g above the knee. He was unable to detect the 128 Hz tuning fork at the great toe or the lateral malleolus, but it was intact at the knee bilaterally.

**Figure 1:**
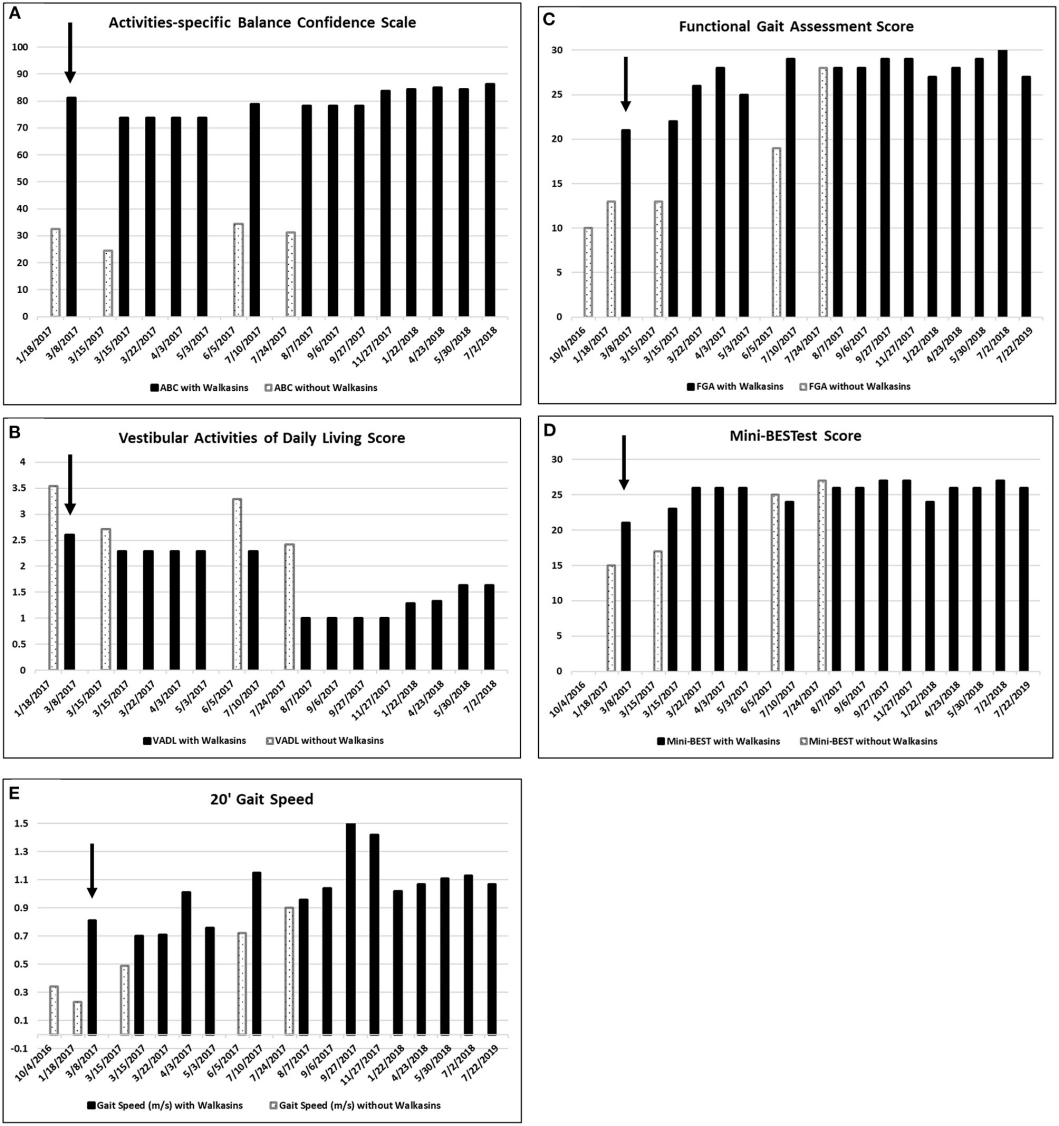
**(A)** Scores on the Activities-specific Balance Confidence (ABC), **(B)** the Vestibular Activities of Daily Living (VADL), (the VADL and ABC were not administered on 7/22/2019), **(C)** Functional Gait Assessment (FGA), **(D)** Mini-BESTest, and **(E)** 20′ Gait Speed prior to and while wearing the Walkasins®. Higher on the ABC, FGA, Mini-BESTest, and 20′ Gait speed and lower scores on the VADL are better. He started wearing the Walkasins® on 3/8/2017 noted with an arrow ↓. Note that occasionally the Walkasins® device was turned off for a day and on those dates his perception of balance and functional ability decreased.

**Figure 2:**
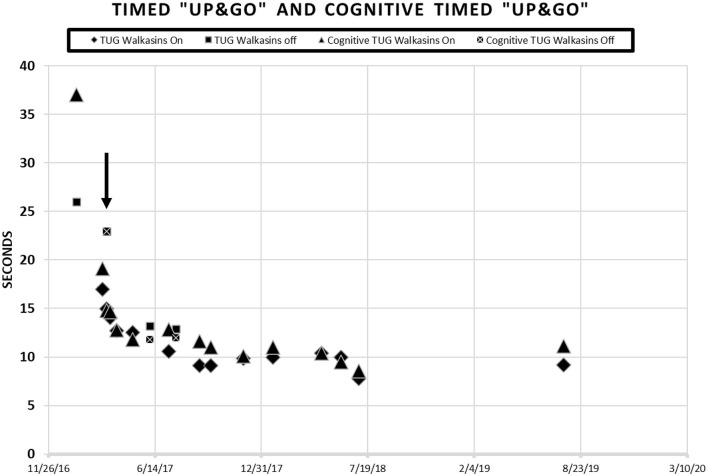
Patient scores on the Timed “Up & Go” and the Cognitive Timed “Up & Go” in seconds prior to and during intervention with the Walkasins®. He started wearing the Walkasins® 3/8/17 as indicated by the arrow ↓. Note some days were tested while wearing the device and some were not. When the tests were performed without the Walkasins®, the patient had not used them for 1 day.

Due to the results of the systems review and observation of his gait in the clinic, the following special tests were completed; The Activities-specific Balance Confidence Scale (ABC) (38), The Vestibular Activities of Daily Living Scale (VADL) (39), 20′ gait speed, Functional Gait Assessment (FGA) (40), miniBESTest (41), Timed “Up & Go” (TUG) (42), and cognitive TUG (43). The results of his initial scores on these tests are illustrated in Figures 1, 2. Although he was seen for 6 months prior to using Walkasins, multiple baseline values were not collected except for 2 measures for the FGA.

The Activities-specific Balance Confidence (ABC) test is a 16 item self-efficacy questionnaire that quantifies how confident people are in their ability to balance during the activities (38). The ABC is scored on a mean with scores ranging from 0 to 100 with higher scores indicating more confidence. The ABC is reliable and valid (38, 44–47). The minimal detectable change (MDC) of the ABC is reported to be 11–15 points depending on the population (44, 45, 48). The ABC has not been evaluated in people with PN. The patient's score on the ABC prior to intervention was 32%.

The Vestibular Activities of Daily Living Scale (VADL) was developed to quantify a person's ability to perform daily activities and perception of assistance needed during functional activities that challenge balance (39). It contains 28 items ranging from bed mobility to driving a car. The VADL was developed to be scored on a median so that an item can be skipped without affecting the score. We scored it on both a median and mean to increase its responsiveness. The reliability and validity of the VADL has been demonstrated in subjects with vestibular dysfunction although an MDC has not been established (39, 49). The patient's mean score on the VADL prior to intervention was 3.5.

Gait speed was calculated on a 20′ walkway with ~4′ acceleration and deceleration space. Calculation of gait speed is considered reliable and valid (50–52). A gait speed of 1.2 m/s is necessary to cross the street (53). The MDC for gait speed is 0.09 m/s for people with Parkinson Disease (51) and for those with Alzheimer's Disease (52), and 0.109 m/s in older adults (54). The patients preferred gait speed was 0.21 m/s prior to intervention. He ambulated with a straight cane in the community.

The Functional Gait Assessment (FGA) was initially developed to quantify gait tasks in people with vestibular dysfunction (55). It includes 10 tasks, including gait at normal, slow and fast speeds, gait with head turns, turning, stepping over obstacles, walking tandem, eyes closed, backwards, and stairs, rated on an ordinal scale. It has been shown to be reliable and valid in many populations (40, 56–63). The MDC of the FGA has been calculated to be 4 points in people with Parkinson's Disease (59) and 6 points in people with vestibular disorders (58). The Minimal Clinically Important Difference in community dwelling older adults is 4 (64). Scores of <22/30 indicate increased fall risk in community dwelling older adults (61). The patient's FGA score prior to intervention was 13/30.

The mini-BESTest was developed to guide treatment for balance disorders. It is a revision of the BESTest to decrease the time for administration. It contains 14 items scored on an ordinal scale of 0–2. It has been shown to be reliable and valid in various populations (65–67). Scores of <16 indicate increased risk of falling in older adults (67). The MDC for the mini-BESTest is 3.5 points in people with balance deficits (65). The patient's mini-BESTest score prior to intervention was 15/28.

Timed “Up & Go” (TUG) is the time it takes a patient to stand from sitting in a chair, walk 3 meters, turn, walk back to the chair and sit down (42). Times >11 s indicate increased risk of falls in older, community dwelling older adults (40, 42, 68, 69). The MDC for the Timed “Up & Go” is 1 s in people with Type II Diabetes (25). The cognitive Timed “Up & Go” (cTUG) requires that a cognitive task such as counting backwards by 3's is performed while completing the test. A 10% difference between scores on the TUG and the cTUG is considered abnormal (41, 70, 71). His TUG score was 26 s, and his cognitive TUG was 37 s prior to intervention.

### Intervention

The Walkasins® is a sensory prosthesis that is intended to replace nerve function used to detect and signal balance related foot pressure sensation that is lost in patients with PN. It is indicated for use by individuals with lower limb sensory peripheral neuropathy who present with gait and balance impairments and are at high risk of falls (36, 37). Pressure sensors embedded in shoe inserts measure plantar pressure, an embedded microprocessor and software estimates center of pressure parameters and activates a relevant tactile actuator around the ankle just above the malleoli providing directional specific information on center of pressure sway to help indicate the position of the body in space. During walking, relevant events of the gait cycle are signaled including heel strike and toe off allowing identification of the stance and swing phases of gait. The device is currently being investigated in a multi-site long-term clinical trial in a cohort of patients with PN (walk2Wellness, ClinicalTrials.gov #NCT03538756). The findings of the first 10 weeks in this trial of Walkasins has demonstrated improvement in gait and balance function in 45 adults with PN (37). Previous short -term studies (31) including a recent in clinic randomized cross-over trial showed short-term improvements in gait and balance function (37).

The patient in the current study had been participating in a student run, pro bono neurological wellness program for 2 times a week over a period of 5 months and was receiving gait and balance training prior to the intervention with the Walkasins®. Although he had seen some improvement in his gait and balance, he was no longer improving. FGA scores increased from 10 in October to 13 in January (Figure 1C). Based on this he was still at an increased risk of falls (61). He reported decreased visual acuity, due to macular edema, in addition to the peripheral neuropathy, so a decision was made to try the Walkasins® to evaluate its use to maximize his function. The use of the device was approved by the Research Review Board at Wingate University. The patient was fitted with the Walkasins® and was instructed in how to use the sensory information for balance by swaying with the eyes open and closed as well as walking. Despite his loss of sensory function below the knee, he was able to feel the vibration from Walkasins and use the information, therefore it was agreed that a trial of the device was warranted. The patient was able to use the information immediately and improvements in gait and balance measures were noted over time (see Figures 1, 2). The patient wore Walkasins® for more than 2 years while participating in the Neurological Wellness Program sporadically. He was aware of his scores on the outcome measures, completed a home exercise program of gait and balance exercises including encouragement to walk in the community, and received positive feedback as he improved.

## Results

The patient's improvements in the gait and balance measures assessed before and during the intervention are shown in Figures 1, 2. Occasional testing occurred with the device turned off. On those occasions, the Walkasins® had been turned off only for that testing day to see if there was carry over. It should be noted that the patient improved his scores beyond the MDC and beyond the fall risk cutoff for each of the outcome measures within the 1st week of using the device. Surprisingly, the improvements seen with the device in gait and balance appeared to be maintained with the device turned off for 1 day following 3–4 months of initial use. The patient's perception of his balance abilities was substantially less when the device was turned off. From a qualitative perspective, the patient reported that the device was easy to use and had greatly improved his balance. He reported that not only had his balance improved but his leg pain and cramping had decreased. He had increased his community ambulation from <0.25 miles to over 5 miles. In addition, he had started running down the hallway in the clinic with supervision.

## Discussion

Improvements were seen immediately following donning the Walkasins® and the improvements continued throughout the 2 years follow-up while participating in the exercise program. The patient continues to wear the Walkasins® and maintain improvements, however only the data from the 2 years is presented here. Improvements in outcomes were maintained even with the device turned off for 1 day. The most plausible explanation for why he improved was that the Walkasins® provided sensory information from his insensate feet and transferred the information to an area of intact sensation around his lower leg. It may also be that the information from the device allowed the brain to align and use other sensory inputs to provide a reference of verticality. Similar changes in gait and balance have been documented in our previously published paper on the results of a 10-week trial. This case report demonstrates continued improvements and benefits of using Walkasins. However, the patient in this study demonstrated greater improvements than those in the other study and demonstrated continued improvements as he participated past 10 weeks, in fact his performance peaked between 4 and 6 months (Figures 1, 2).

It was unexpected that the Walkasins® may have played a role in decreasing pain and muscle cramping. The mechanism of this is unclear but it is thought that the new relevant sensory information regarding foot pressure provided from Walkasins® may block the allodynia seen with peripheral neuropathy. What is surprising and harder to explain is that the pain relief continued during periods of the day in which the patient was not wearing the device although the increased level of physical activity by the patient may in itself improve his overall health and fitness leading to less pain.

Another unexpected observation was that the gait and balance improvements were seen even when the device was turned off for a day. It may be that the brain learned to use other sensory inputs for postural control. The patient's perception of his balance abilities decreased as soon as the device was turned off. A possible explanation of the improvements seen with the Walkasins® was due to a placebo effect due to improved confidence. However, this does not seem likely due to his physical performance with the device while turned on. He did know whether the device was on or off during testing as he could tell when it was working. Future research should be conducted to determine what would happen if the device would be turned off for longer periods of time. Since the Walkasins® neuroprosthesis attempts to substitute for lost foot pressure sensation required for balance, it may be hypothesized that overall balance function will deteriorate with the device off.

The patient continued to participate in the Neurological Wellness Program while wearing the Walkasins®, making it difficult to determine whether the gait and balance training or wearing of the Walkasins® made the difference in the outcome measures. It is unlikely that the Neurological Wellness Program made that dramatic of a change as he had participated in the program for 5 months before he used the Walkasins®, and since his progress had plateaued. Further research is needed to determine the effect of the Walkasins® on gait and balance in persons with insensitive feet.

## Limitations

There are several limitations of this study: (1) This is a case report so the findings cannot be generalized. (2) The subject continued to participate in a Neurological Wellness Program so the changes may have been due to his continued interventions. (3) The patient was highly motivated, other patients without high motivation may not see such improvements. (4) The patient was relatively young compared to many people with peripheral neuropathy, his improvements may have been greater due to his age.

## Conclusions

The use of the Walkasins® appears to have led to improved gait, balance, and self-perceived stability in a patient with diabetic PN. Using sensory substitution devices may be a new intervention technique for people with gait and balance dysfunction due to PN. Additional long term clinical trials are needed to determine whether this is true and generalizable to the population of people with peripheral neuropathy. Further studies are also needed to determine the optimum residual sensory function needed to use the Walkasins® and the need for gait and balance training simultaneously.

## Data Availability Statement

The original contributions presented in the study are included in the article/supplementary material, further inquiries can be directed to the corresponding author/s.

## Ethics Statement

The studies involving human participants were reviewed and approved by Wingate University Research Review Board. The patients/participants provided their written informed consent to participate in this study. Written informed consent was obtained from the individual(s) for the publication of any potentially identifiable images or data included in this article.

## Author Contributions

DW and LO concept, data analysis, writing, and approval of manuscript. DW provided intervention, data collection, and project coordination. LO RxFunction supplied Walkasins. GM and JH provided intervention, data collection, and approval of manuscript. All authors contributed to the article and approved the submitted version.

## Conflict of Interest

Walkasins was supplied by RxFunction. LO is co-inventor of the Walkasins technology and co-founder of RxFunction, Inc., the medical device startup that is commercializing Walkasins. LO is currently employed as CTO of the company, is a shareholder in the company, and a member of the Board of Directors. The remaining authors declare that the research was conducted in the absence of any commercial or financial relationships that could be construed as a potential conflict of interest.
